# Transplantation of 3D adipose-derived stem cell/hepatocyte spheroids alleviates chronic hepatic damage in a rat model of thioacetamide-induced liver cirrhosis

**DOI:** 10.1038/s41598-022-05174-2

**Published:** 2022-01-24

**Authors:** Yu Chiuan Wu, Guan Xuan Wu, Kuan Wei Chen, Li-Yen Shiu, Satheesh Kumar, Guei-Sheung Liu, Shyh Ming Kuo

**Affiliations:** 1 Hualien Armed Forced General Hospital, Hualien, Taiwan; 2 National Kaohsiung University of Hospitality and Tourism, Kaohsiung City, Taiwan; 3 Ministry of National Defense-Medical Affairs Bureau Medical Planning Division, Taipei City, Taiwan; 4 Department of Biomedical Engineering, I-Shou University, Kaohsiung City, Taiwan; 5 Cell Therapy Center, E-Da Hospital, Kaohsiung City, Taiwan; 6 Menzies Institute for Medical Research, University of Tasmania, Hobart, TAS Australia; 7 Centre for Eye Research Australia, Royal Victorian Eye and Ear Hospital, East Melbourne, VIC Australia; 8 Ophthalmology, Department of Surgery, University of Melbourne, East Melbourne, VIC Australia

**Keywords:** Biotechnology, Biomaterials, Biomaterials - cells

## Abstract

Cirrhosis refers to irreversible liver damage where healthy tissue is replaced by scar tissue, resulting in impaired liver function. There is no cure and current treatments only prevent further liver damage; thus, novel therapeutic options are urgently needed. Here, we report a new approach that enables the formation of self-assembled 3D spheroids of adipose-derived stem cells (ADSCs) and murine hepatocytes (AML12) via reconstituted collagen fibers. Compared with the spheroids formed in the commercially available EZSHERE dish, the collagen fiber-based ADSC/hepatocyte spheroids offer a notable benefit in structure formation and paracrine factor secretion. To test the regenerative capability of the collagen fiber-based 3D ADSC/hepatocyte spheroids, a rat model of thioacetamide (TAA)-induced liver cirrhosis was employed. The transplantation of the collagen fiber-based 3D ADSC/hepatocyte spheroids show an improvement in liver function and ameliorates pathological liver cirrhosis in TAA-treated rats. In summary, our data show collagen fiber-based self-assembled 3D ADSC/hepatocyte spheroids to possess the excellent regenerative capacity in response to TAA-induced liver injury, promising an alternative therapeutic strategy for liver cirrhosis.

## Introduction

The liver is the largest organ inside the human body and is capable of self-regeneration under normal physiologic conditions. The liver is also involved in many essential functions; for instance, it detoxifies blood, fights against infections, and aids digestion. Therefore, liver diseases severely affect overall human health. Long-term damage to the liver results in the gradual loss of liver function and accumulation of the extracellular matrix (ECM). Eventually, this leads to liver cirrhosis, histologically characterized by fibrous septa dissecting the liver parenchyma. Cirrhosis is a severe and advanced stage of chronic liver disease that frequently progresses to hepatocellular carcinoma (HCC). To date, liver transplantation is the only recommended treatment for liver cirrhosis. However, donor liver shortage, high cost, surgical damage, and lifelong immunosuppression limit the applicability of this treatment modality^1^. Recently, cell therapy has been developed as an effective alternative therapy to organ transplantation because of its minimally invasive nature and considerably fewer complications^2^. Hepatocytes are the principal cells that maintain liver function. In a healthy liver, hepatocytes can proliferate to restore the functional liver mass. In addition, the liver contains progenitor cells that can proliferate and differentiate into hepatocytes^3^. However, these functions are compromised in liver cirrhosis^4^. Therefore, effective therapies must be developed to prevent further damage to the liver, promote liver tissue regeneration, and restore liver function.

A major challenge in liver tissue engineering is the functional deterioration of hepatocytes cultured in two-dimensional (2D) monolayer substrates, as it readily introduces critical alterations to the cellular phenotype. Cultured hepatocytes exhibit a flattened and extended shape, a substantial reduction in liver-specific gene expression, and loss of metabolic functions such as albumin secretion and urea synthesis^5^. These findings indicate that an appropriate three-dimensional (3D) microenvironment prepared using various biomaterials or architectures is essential to preserve hepatocyte function. For example, compared with a 2D monolayer culture, hepatocytes exhibit a more normal cuboidal and polarized shape and higher hepatic functionality in a 3D spheroid culture^6^. Spheroid cultures offer a cellular niche that is similar to the tissue-like microenvironment^7^. Several approaches including the nonadherent surface culture, dynamic bioreactor culture, and hanging-drop method have been used to produce spheroid cultures^6^. A spheroid culture is a typical scaffold-free 3D cell culture system that either forces cell self-assembly or induces cell clusters from cell suspensions^8^. Although these approaches can produce large amounts of uniform spheroids, the lack of necessary ECMs for cells can cause long-term impairment of cellular functions^9^.

For liver tissue engineering applications, patient-derived hepatocytes were initially considered to be the most favorable cells for treating end-stage liver diseases. However, the use of patient-derived hepatocytes is time-consuming, and adequate donors and immunosuppressive agents are required. Mesenchymal stem cells (MSCs)-based regenerative therapy has gained attention as an effective alternative treatment for liver diseases due to the ability of MSCs to repair damaged tissues and restore original function by differentiating into target cells^10^. Numerous studies have also focused on using adipose-derived stem cells (ADSCs), extracted from subcutaneous fat tissues during a liposuction procedure. The properties of ADSCs, including their differentiation potential and favorable angiogenic and anti-inflammatory cytokine secretion profile, appear comparable to MSCs from other source organs. Thus, ADSCs are being widely used in tissue engineering and clinical regenerative medicine^11^.

In the present study, we have developed a new self-assembly method that enables formation of 3D ADSCs/hepatocyte spheroids, using reconstituted collagen fibers. We investigated the fate and functions of the collagen fiber-based self-assembled 3D spheroids in comparison with spheroids formed by a commercially available EZSHERE dish method. Therapeutic efficacy of the 3D spheroids was further evaluated on a well-established rat model of thioacetamide (TAA)-induced liver cirrhosis.

## Results and discussion

### Characterization of ADSCs and bio-manufacturing of collagen fiber-based 3D spheroids

**Figure 1:**
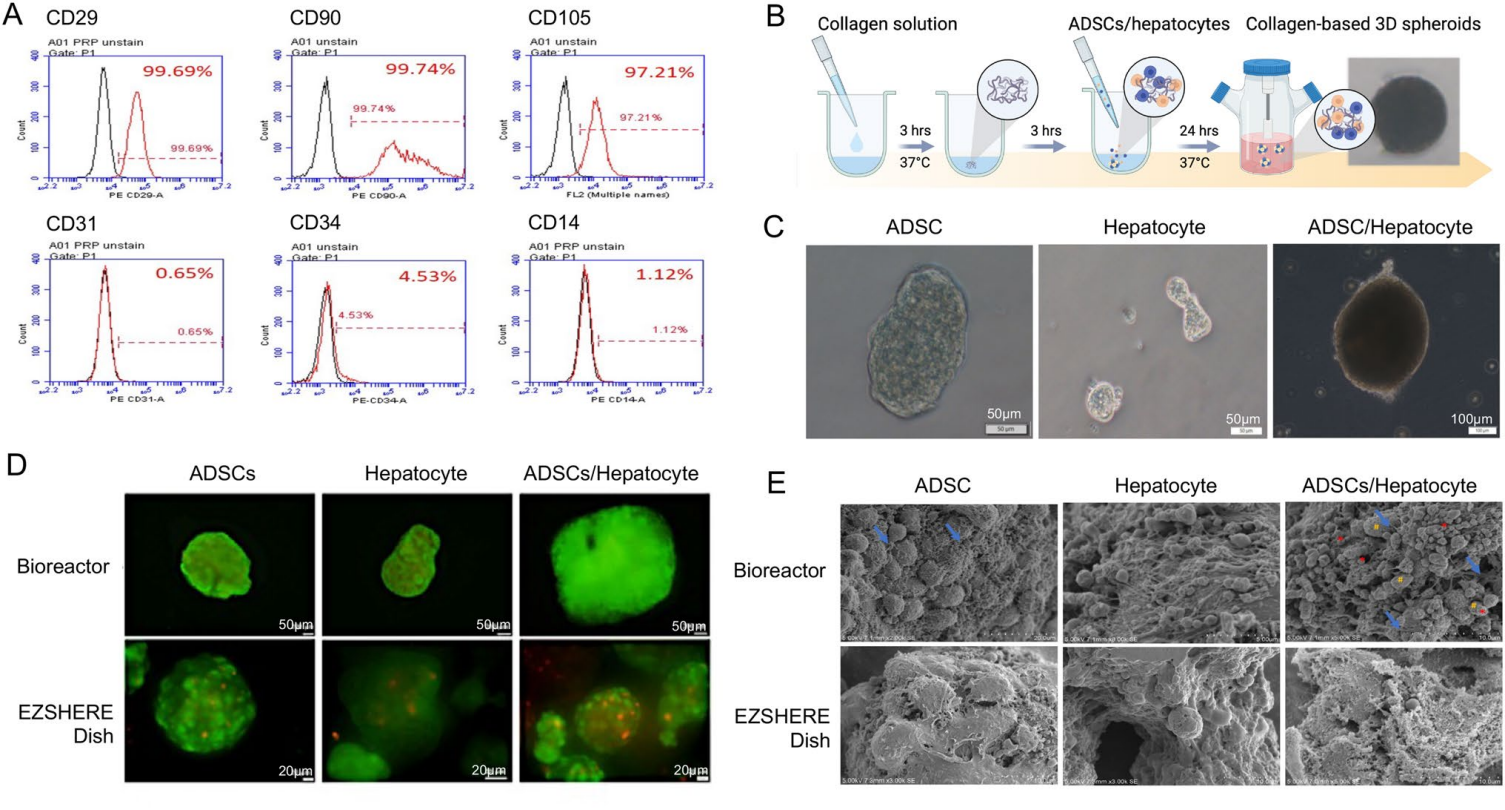
(**A**) Expression of CD29, CD90, CD105, CD31, CD34, and CD14 in isolated adipose-derived stem cells (ADSCs). (**B**) Schematic of the production of cell spheroids. (**C**) The produced spheroids in the bioreactor (after 24 h culture). Scale bar: 50 or 100 μm. Created with BioRender.com. (**D**) Live/dead fluorescence assay of spheroids. Green: calcein-AM and red: EthD-1. Scale bar: 50 μm (top panels) and 20 μm (bottom panels). (**E**) Scanning electron microscopy (SEM) images of ADSCs and hepatocytes cultured in spheroids produced in the bioreactor. Blue arrow: collagen fibers, red*: hepatocytes, and yellow#: ADSCs.

ADSCs were characterized using flow cytometry. As shown in Fig. 1A, cells isolated from the adipose tissue expressed a high percentage of ADSC markers, namely CD29 (99.69%), CD90 (99.74%), and CD105 (97.21%), and a low percentage of hematopoietic markers, namely CD31 (0.65%), CD34 (4.53%) and CD14 (1.12%).

Reconstituted collagen fibers were used as an adherent substrate to generate 3D spheroids in the bioreactor. Similar to the established 3D spheroids formation approach using the commercially available EZSHERE dish (REPROCELL USA Inc), the collagen fiber-based methodology also generates 3D spheroids (ADSC, hepatocytes, and ADSC/hepatocytes hybrid 3D spheroids) within 24 h (Fig. 1B,C). The size of spheroids produced using our approach was considerably larger than those produced from the EZSHERE dish (Table 1). We also found that the spheroids exhibited lower sphericity due to continuous stirring during the preparation procedure. Among all spheroids, the size of hepatocyte spheroids was the largest (826.3 ± 350.6 μm); this increase in size might be attributed to the polygonal shape of hepatocytes and the absence of pseudopodia firmly attached onto collagen fibers. In contrast, cells were aggregated tightly and yielded substantially smaller-sized spheroids in the static EZSHERE dish (Table 1). In addition, the size of spheroids formed by the collagen fiber-based approach increased gradually over the culture period of 72 h. However, the spheroids started to degrade, spheroids broke, and debris of smaller and irregular shape spheroids was observed after 72 h (data not shown). Figure 1D shows the cell viability in the formed spheroids from a live/dead fluorescence assay. Lesser red colored (dead cell) stains were observed in the spheroids produced from bioreactor that using reconstituted collagen fibers as ingredients/ECM component, which benefits cell adhere and survive. Notably, scanning electron microscopy images showed the collagen fiber network closely bound to ADSCs and hepatocytes, maintaining the structure of spheroids (Fig. 1E). These data suggest that the collagen fiber-based approach has remarkable benefits for growing and maintaining spheroids.

**Table 1:** Comparison of the spheroids generated from collagen fiber-based method and EZSHERE dish.

Culture vehicle	Inoculated cells	Ease of stable spheroid formation			Sphericity	Spheroid size (μm) 72-h culture
		24-h culture	48-h culture	72-h culture		
Collagen fiber/bioreactor	ADSCs	++	++	+++	SS	437 ± 96
	hepatocytes	+	+	++	S	826 ± 350
	ADSCs/hepatocytes (1:1)	++	++	++	SSS	499 ± 64
EZSHERE dish	ADSCs	++	++	+++	SSS	248 ± 53
	hepatocytes	++	++	++	SS	168 ± 37
	ADSCs/ hepatocytes (1:1)	++	++	+++	SSS	151 ± 32

+ : ease, more + means more ease; × : cannot form; S: sphericity, more S means more sphericity.

### Collagen fiber-based 3D spheroids maintains cell-specific characteristics and enhances paracrine factors secretion

**Figure 2:**
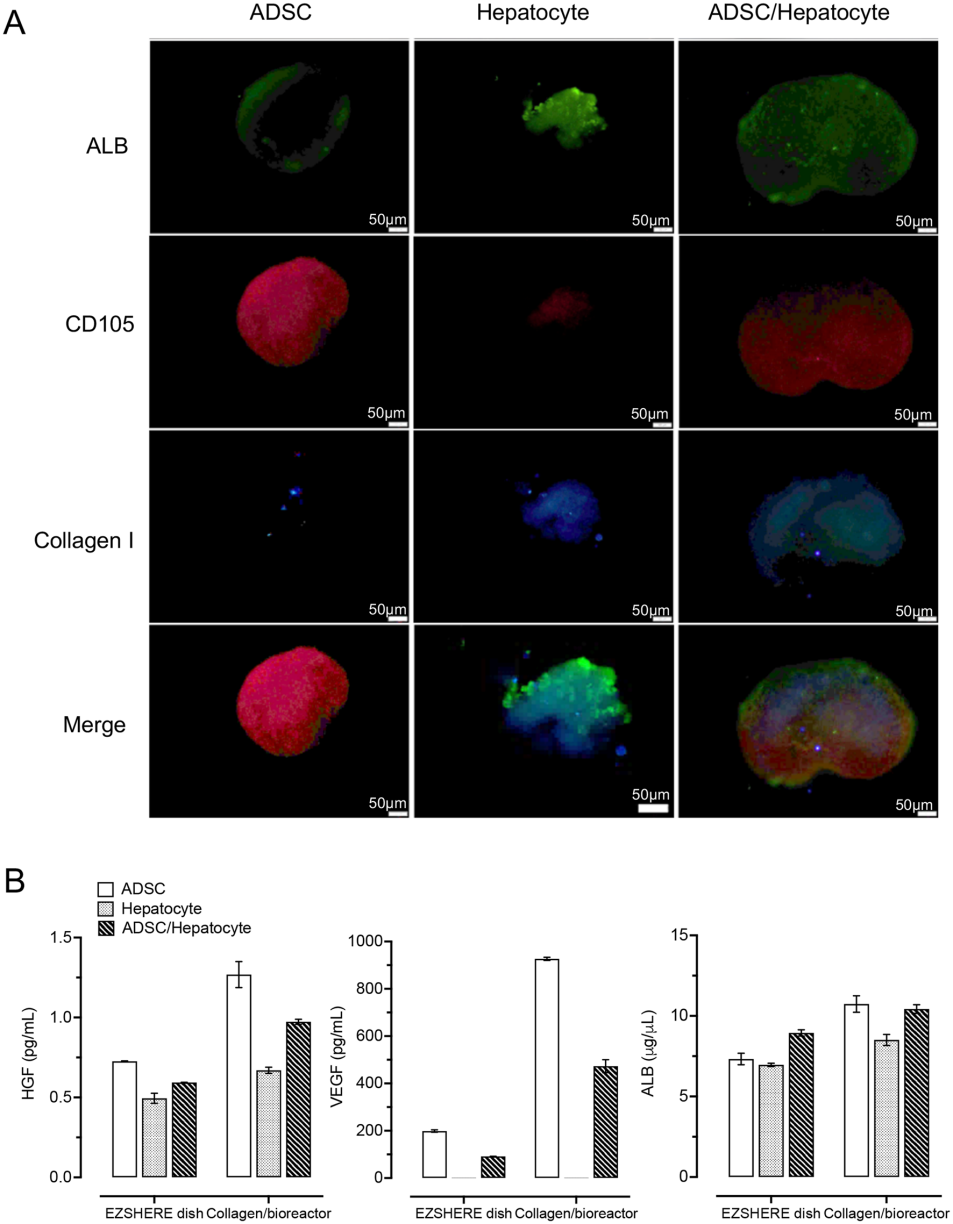
(**A**) Fluorescence images of spheroids. Albumin (hepatocyte marker, green), collagen I (hepatocyte marker, blue) and CD105 (ADSC marker, red) were visualized at the 3 days of culture. Scale bar: 50 μm. (**B**) Hepatic growth factor (HGF), vascular endothelial growth factor (VEGF) and albumin secreted by adipose-derived stem cell (ADSC) and hepatocyte spheroids produced by the collagen fiber/bioreactor-based approach and EZSHERE dish (2-day culture). Group data are shown as means ± SEM.

Preserving function of cultured cells is crucial for cell therapy. A major challenge in liver tissue engineering is the functional deterioration of hepatocytes in vitro. Approaches such as sandwich culture and spheroid culture have been developed to preserve hepatocyte function. Studies have reported that the addition of growth factors such as vascular endothelial growth factor (VEGF), hepatocyte growth factor (HGF), and platelet-derived growth factor (PDGF) could prevent the loss of hepatocyte-specific characteristics^12^. Figure 2A shows the expression of albumin (ALB), collagen I and CD105 in collagen fiber-based 3D spheroids after 48 h. We found that ALB, collagen I and CD105 are strongly expressed in mouse hepatocytes and ADSC spheroids indicating cell-specific characteristics of hepatocytes and ADSCs to be well maintained when 3D spheroids are formed. Recent studies have reported that HGF and VEGF secreted by ADSCs appear to substantially promote hepatocyte proliferation and survival^13^. We evaluated HGF and VEGF levels secreted from collagen fiber-based 3D spheroids. A significant increase in VEGF secretion was found in ADSC and ADSC/hepatocyte 3D spheroids formed by collagen fibers compared to those formed in the EZSHERE dish (Fig. 2B). Similar findings were also observed in HGF and ALB secretions from collagen fiber-based 3D spheroids (Fig. 2B). Overall, our data indicates that the collagen fiber-based 3D spheroids present a notable benefit in paracrine factors secretion, suggesting they may help in maintaining hepatocyte function.

### Transplantation of 3D adipose-derived stem cell/hepatocyte spheroids alleviates fibrotic response in TAA-induced liver cirrhosis

**Figure 3:**
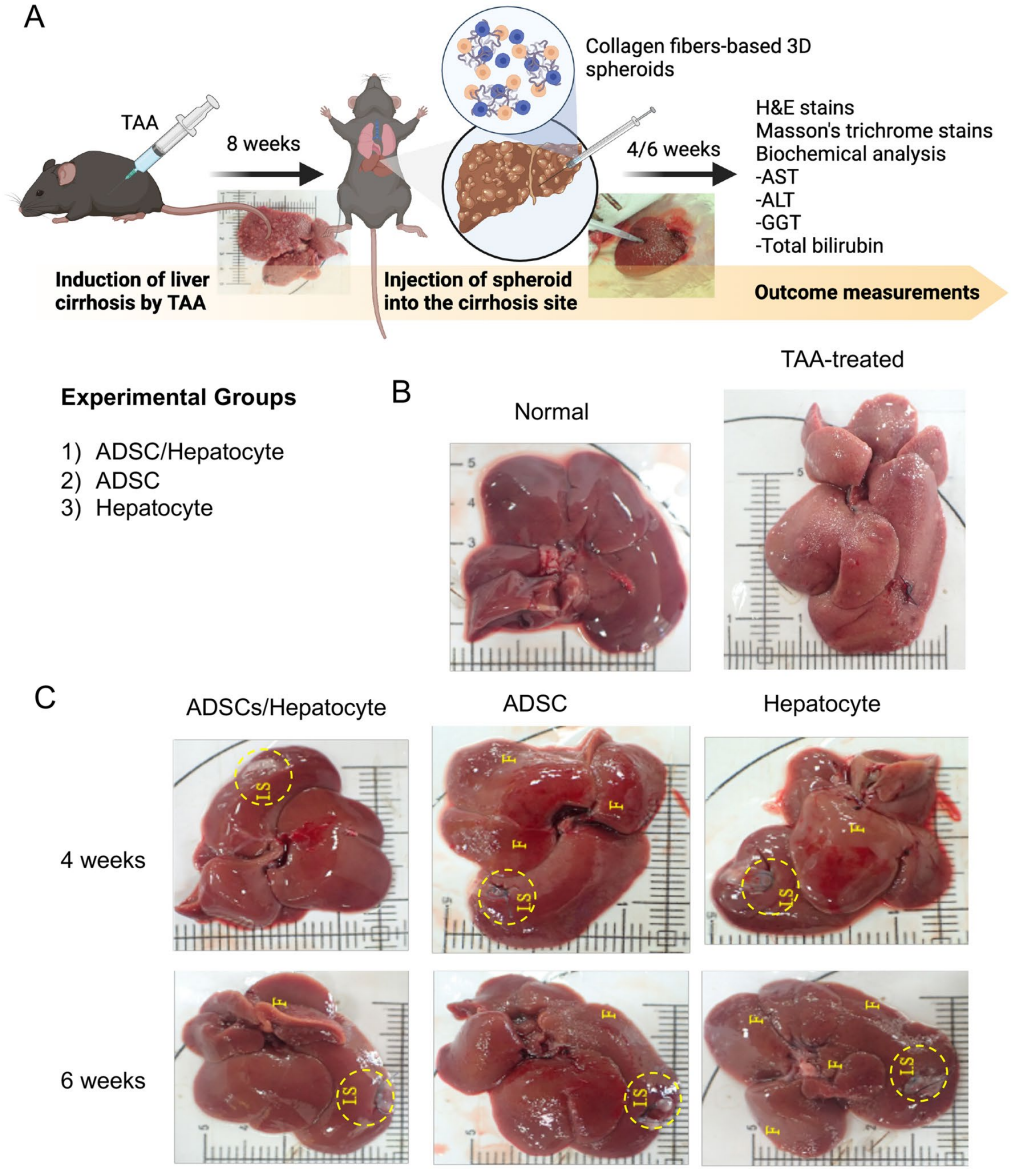
(**A**) Experimental design of in vivo study. Created with BioRender.com. (**B**) Gross observation of thioacetamide (TAA)-induced liver cirrhosis and experimental groups. Severe liver fibrosis caused regenerating nodules in the TAA-induced liver cirrhosis without treatment. (**C**) ADSC/hepatocyte spheroids: regenerating nodules were not seen, and fibrotic structures were only found at the margin of a few lobes at week 4, whereas fibrotic structures were barely seen at week 6. ADSC spheroids: fibrotic structures were found in a lobe with a larger area at week 4 but fibrotic areas were enlarged at week 6. hepatocyte spheroids: fibrotic structures were observed in several lobes of the liver at weeks 4 and 6. IS, implantation site; F, fibrous.

To evaluate regenerative function of the collagen fiber-based 3D spheroids, a rat model of TAA-induced liver cirrhosis was employed (Fig. 3A). Compared with carbon tetrachloride, TAA has been reported to reliably induce liver cirrhosis in vivo^14^. Regenerating nodules caused by severe liver fibrosis were observed in the rats with TAA-induced liver cirrhosis compared to the healthy liver with a smooth surface in control rats (Fig. 3B). Figure 3C shows the gross pathological observations of the rat livers of each experimental group after TAA-induced liver cirrhosis. We observed a remarkable decrease in liver fibrosis (found only at the margin of a few lobes) in the rats that received the ADSC/hepatocyte spheroids 4 and 6 weeks after implantation. In the rats that received collagen fiber-based 3D ADSC spheroids, the fibrotic structures were slightly decreased and still found in the lobe at 4 and 6 weeks after implantation. Moreover, a severe fibrotic structure in several lobes was observed in the rats that received collagen fiber-based 3D hepatocyte spheroids at 4 and 6 weeks after implantation, indicating the low regenerative capacity of these spheroids.

**Figure 4:**
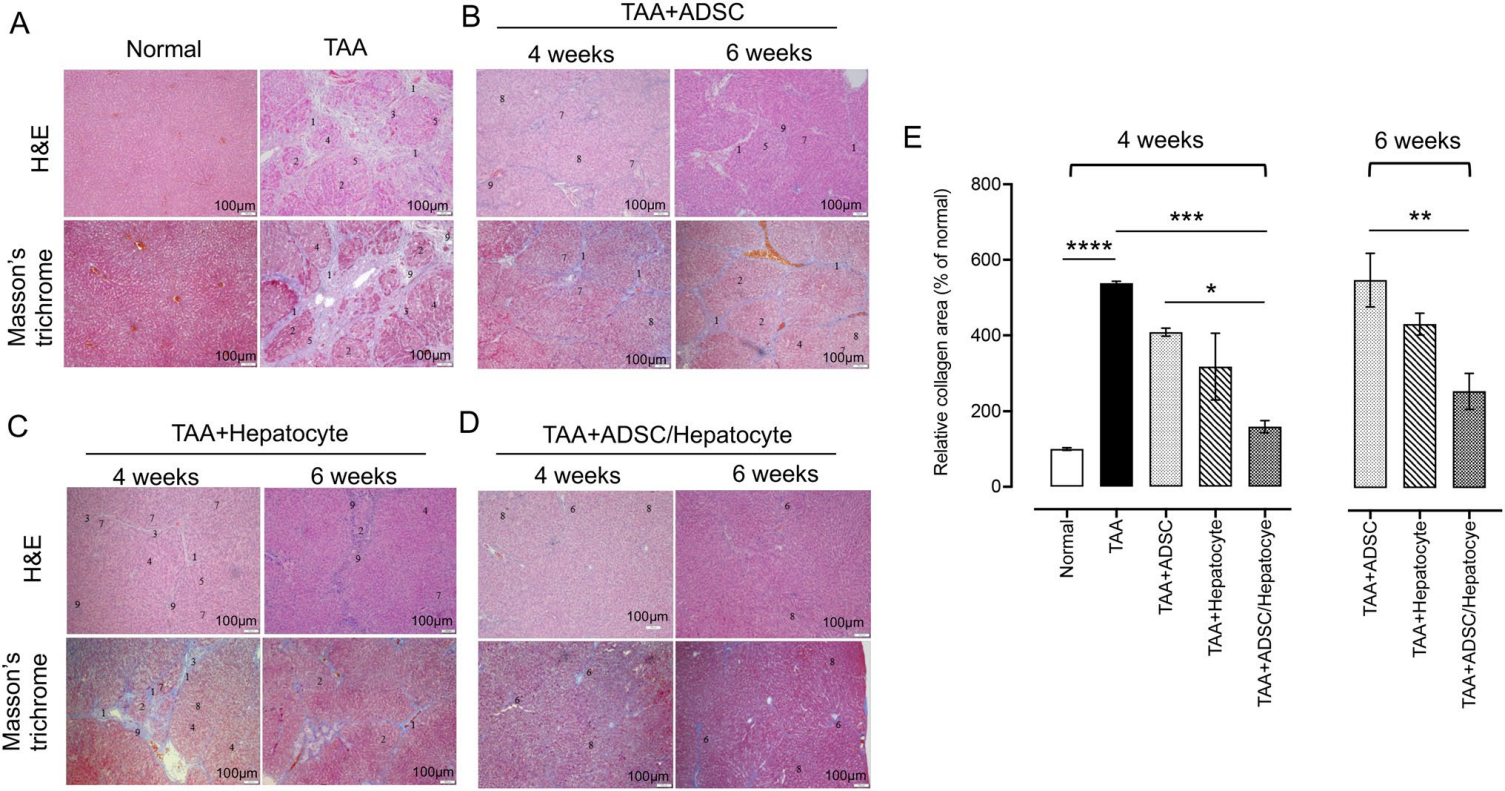
Histological analysis of thioacetamide (TAA)-induced liver cirrhosis in the various experimental groups. (**A**) In rats with TAA-induced liver cirrhosis (n = 3), the severe fibrotic pathological features were observed by hematoxylin and eosin (H&E) and Masson’s trichrome staining. (**B**–**D**) In rats treated with ADSC, hepatocyte or hybrid ADSC/hepatocyte, spheroids reveal a beneficial effect on alleviating the severe fibrotic pathological features in TAA-induced liver cirrhosis. Scale bar: 100 μm. (**E**) Quantitative analysis of the amount of collagen stained by Masson's trichrome in each group. The description of pathological features: (1) reticular fibers circumscribing the nodules; (2) obvious formation of pseudolobules; (3) numerous fibrocytes; (4) hepatocyte necrosis; (5) swollen hepatocytes; (6) large/moderate decreases in fibrous tissues/fibrocytes; (7) proliferation of the mini-bile duct; (8) fatty degeneration-like areas; (9) inflammatory cell infiltration. Group data are shown as means ± SEM. Statistical analysis was undertaken with One-way ANOVA; *P < 0.05; **, *P* < 0.01; ***, *P* < 0.001, ****, *P* < 0.00001.

Histopathological analysis was performed to evaluate regenerative function of collagen fiber-based 3D ADSC/hepatocyte spheroids in the cirrhotic livers. The fibrotic response of cirrhotic livers was assessed in rats by hematoxylin and eosin (H&E) staining and Masson's trichrome staining. In comparison to the healthy liver in control rats, severe reticular fibrotic tissues were clearly observed in the regenerating nodules of cirrhotic livers in rats that had received TAA treatment for 4 weeks (Fig. 4A). Four and six weeks after collagen fiber-based 3D ADSC/hepatocyte spheroids implantation, the level of fibrotic response in the rat livers with treatment was less than in rats without treatment (Fig. 4D,E). There was a slight to moderate decrease in the reticular fibers circumscribing the regenerating nodules in the rats that had received either collagen fiber-based 3D ADSC or hepatocyte spheroids compared with rats that had not received the spheroid implantations (Fig. 4B,C,E). Our results indicate that the collagen fiber-based 3D ADSC/hepatocyte spheroids exhibit stronger regenerative effects that modulate the fibrotic response after chronic liver damage. The data is also in accordance with previous results that suggest cellular communication and guidance between ADSCs and hepatocytes, which is beneficial for stem cells to exert their reparative effects on liver fibrosis and cirrhosis.

### Transplantation of 3D adipose-derived stem cell/hepatocyte spheroids preserves liver function in TAA-induced liver cirrhosis

**Figure 5:**
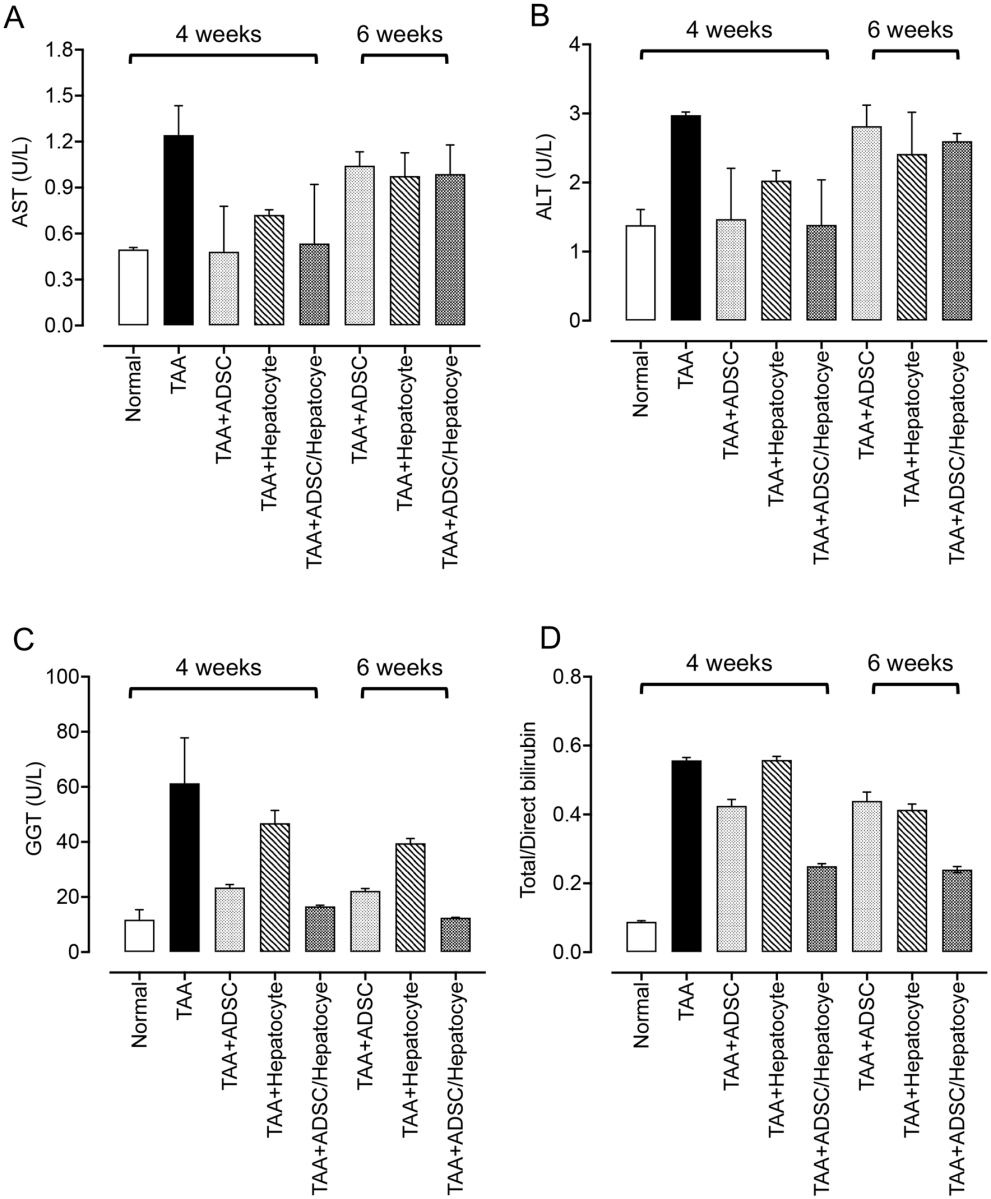
Serum biochemical data of (**A**) aspartate aminotransferase (AST), (**B**) alanine aminotransferase (ALT), (**C**) γ-glutamyltransferase (γ-GT), and (**D**) total/direct bilirubin in the experimental groups (n = 2 from 4 rats). Group data are shown as means ± SEM.

Alteration in serum levels of AST, ALT, and γ-GT (GGT) have been widely used as markers to indicate liver function and prognosis of liver diseases^15^. TAA-induced liver damage has been shown to increase serum levels of AST, ALT, and total bilirubin in rats^16^. Higher bilirubin levels usually indicate injury to liver cells or bile duct, and gallbladder obstruction that causes inefficient elimination of bilirubin by the liver. We therefore measured serum levels of AST, ALT, GGT and total/direct bilirubin to evaluate liver function in the TAA-treated rats that received the collagen fiber-based 3D spheroid treatment. A reduction in the serum levels of AST, ALT, GGT and total/direct bilirubin was observed in the rats that had received the collagen fiber-based 3D ADSC/hepatocyte spheroids at weeks 4 after implantation (Fig. 5), suggesting improvement of liver function in TAA-treated rats. However, the levels of AST and ALT rebounded at week 6, indicating reparative effects of collagen fiber-based 3D ADSC/hepatocyte spheroids in some areas of the liver may somewhat decline when treatment lasts over 4 weeks. Interestingly, we also observed a moderate reduction in serum levels of AST, ALT, GGT and total/direct bilirubin in the rats that had received the collagen fiber-based 3D ADSC spheroids at weeks 4 after implantation. These results indicated that ADSC-based therapy could also improve liver function, however without the guidance of hepatocytes, the beneficial effect may be limited and only exist for a short period of time. No beneficial effect in liver function was observed in the rats that had received the collagen fiber-based 3D hepatocyte spheroids, indicating that single liver cell spheroid therapy did not exert any therapeutic effect on liver cirrhosis.

### Conclusion

The aim of liver tissue engineering is to reproduce a functional organ for the treatment of acute and chronic liver disorders. Researchers have attempted to replace liver transplantation with liver tissue constructs developed in vitro. Our study demonstrated that the collagen fiber/bioreactor-based approach eased the production of cell spheroids and provided a notable benefit in structure formation and paracrine factor secretion. We further showed that hybrid ADSCs/hepatocyte spheroids structured by collagen fibers consistently exhibited a beneficial effect in ameliorating fibrotic and potentially preserving liver function in TAA-induced liver cirrhosis rat models. Based on our findings, a second injection of hybrid ADSCs/hepatocyte spheroids may be required for the therapeutic effect to last longer than 6 weeks. Overall, our study shows that collagen fiber-based 3D ADSC/hepatocyte spheroids possess an excellent regenerative capacity in response to TAA-induced liver injury and could potentially be an alternative therapeutic strategy for liver cirrhosis.

## Materials and methods

### Isolation and culture of ADSCs

All cell culture experiments involving human ADSCs were approved by the Human Research Ethics committees of the E-Da Hospital (Kaohsiung, Taiwan) (Ethic No. EMRP51104N) and carried out in accordance with the approved guidelines. Informed consent was obtained from all subjects. Human adipose tissue was harvested from subcutaneous fat obtained during abdominal liposuction surgery. The fat tissues were washed with PBS twice and cut into small pieces (1–2 mm^3^). Subsequently, the tissues were digested with collagenase type I in Hank’s balanced salt solution at 37 °C. Fetal bovine serum was then added, and resultant cells were centrifuged at 800 g for 10 min and collected. The cell pellet was washed with PBS and treated with ammonium-chloride-potassium lysing buffer solution to lyse red blood cells. These cells were centrifuged at 400 g for 10 min and resuspended in Dulbecco’s modified Eagle’s medium supplemented with 10% FBS and 1% penicillin–streptomycin (Thermo Fisher Scientific). The supernatant and debris were removed by changing the fresh medium after 48 h incubation. The purity of ADSCs was characterized by determining surface markers CD29(+), CD105(+), CD90(+), CD31 (−), CD34 (−) and CD14 (−) by using a flow cytometer (Accuri C6; BD, USA).

### Fabrication of collagen fibers-based 3D spheroids

A stirring bioreactor was used to produce the 3D cell spheroids (Fig. 1B). Briefly, collagen monomers (1 mL, 0.01 mg/mL) were reconstituted at 37 °C for 3 h to produce collagen fibers (intended to act both as the adherent and ECM component). 1 mL of 1.5 × 10^5^ cells/mL of ADSCs (passage 2) or mouse hepatocytes (AML12; ATCC, VA, USA) were mixed with the reconstituted collagen fibers and incubated at 37 °C for another 3 h. The collagen fiber-cell mixture was then transferred into the stirring bioreactor (at 40 rpm stirring) at 37 °C to enable stable cell spheroid formation. 3D spheroids (ADSC/hepatocyte spheroids, ADSC spheroids, and hepatocyte spheroids) were harvested after cultivation for a predetermined period. The sphericity and size of spheroids were examined. The 3D spheroids generated by using a commercially available EZSHERE dish were used as control.

### Cell viability assay

The viability of cells in collagen fiber-based 3D spheroids was examined using a live/dead cell assay (Thermo Fisher Scientific). Briefly, an assay solution was prepared by mixing 1 mL of PBS containing 2.5 μL/mL of 4 μM ethidium homodimer-1 (EthD-1) and 1 μL/mL of 2 μM calcein-AM solution. This assay solution (100 μL) was added to the culture and the mixture was placed at 37 °C in 5% CO_2_ for 15 min. The culture medium was removed, and the sample was observed using a fluorescence microscope using excitation filters of 494 nm (calcein-AM) and 528 nm (EthD-1) (Olympus IX71, Japan).

### Albumin and cytokine enzyme-linked immunosorbent assay (ELISA)

To examine the level of albumin and cytokines secreted by 3D spheroids, the medium from cultured samples was collected. The secreted protein level of albumin, vascular endothelial growth factor (VEGF), and hepatic growth factor (HGF) in the collected culture medium were quantified using ELISA kits (BioVision, Milpitas, CA, USA) following manufacturers’ instructions.

### Animals

Animal studies were approved by the Institutional Animal Care and Use Committee of I-Shou University, Taiwan (AUP-ISU-106-50-05) and carried out in accordance with the approved guidelines. The animal studies are reported in accordance and comply with the ARRIVE guidelines. Sprague–Dawley rats (male, 6 weeks old) were supplied by National Laboratory Animal Center (NLAC) Taiwan and housed in standard cages, with free access to food and water in a temperature-controlled environment under a 12-h light (50 lx illumination) and 12-h dark (< 10 lx illumination) cycle.

### Experimental design and cell implantation

Animal experiments were performed over 14 weeks (Fig. 3A). Liver cirrhosis was induced by TAA. TAA working solution (80 mg/mL; Sigma-Aldrich, St. Louis, MO, USA) was prepared with PBS. A total of 19 rats were used to establish the liver cirrhosis rat model and 3 rats served as the normal control. Rats were intraperitoneally injected three doses of 100 μL of TAA solution (80 mg/mL in PBS) per week for a total of 8 weeks. Rats were anesthetized using Zoletil® (intraperitoneal administration of 40 mg/kg of tiletamine with 50 mg/kg of zolazepam,) and xylazine (10 mg/kg), and randomly allocated into the following groups (n = 3/group): Group A- implanted with 3D ADSC/hepatocyte spheroids, Group B- implanted with ADSC spheroids, Group C- implanted with hepatocyte spheroids, and Group D- no treatment, served as a negative control group. Surgery in rats was performed by a surgeon and monitored by experienced veterinarians and 3D cell spheroids were implanted into one lesion site (left lateral lobe; three spheroids/rat). During operation, clinical signs of pain, salivation, or abnormal behavior were carefully monitored^17^. Any issues arising from the injection, resulted in exclusion from the study. Subsequently, randomly selected rats from each group were sacrificed at weeks 4 and 6 after 3D cell spheroids implantation. The liver and serum were harvested for histopathological and biochemical analysis.

### Histopathological analyses

At weeks 4 and 6 post cell spheroids implantation, the rat livers tissues from all groups were harvested and fixed in 10% neutral-buffered formalin. The liver tissues (left lateral lobe) were then dehydrated in graded ethanol solutions, cleared in xylene, embedded in paraffin blocks, and cut into 3 μm-thick sections. H&E staining was performed for the general histopathological examination of the livers. Masson’s trichrome staining was conducted to assess changes in the collagen content of liver tissue. ImageJ software (Version 1.50; National Institutes of Health, USA) was used to measure the collagen content in each group^18^. Three microscopic fields were taken under 100 × magnification from each liver tissue for ImageJ analysis.

### Biochemical assays

Blood biochemical parameters, including alanine aminotransferase (ALT), aspartate aminotransferase (AST), total bilirubin, and γ-glutamyltransferase (γ-GT) were assayed to evaluate rat liver function. Rat serums were isolated from whole blood sample and subjected to ALT, AST, γ-GT and total bilirubin biochemical analysis according to the manufacturer’s instructions.

### Statistical analysis

All values are expressed as the mean ± standard error of the mean (SEM). Comparisons among multiple groups were analyzed by one-way ANOVA followed by Tukey’s multiple comparison. A *p* value of < 0.05 was deemed statistically significant. All statistical analyses were performed using SPSS, version 17.0.

## Data Availability

All datasets generated for this study are included in the article/supplementary materials.
